# The effects of parental opioid use on the parent–child relationship and children’s developmental and behavioral outcomes: a systematic review of published reports

**DOI:** 10.1186/s13034-019-0266-3

**Published:** 2019-01-12

**Authors:** Magdalena Romanowicz, Jennifer L. Vande Voort, Julia Shekunov, Tyler S. Oesterle, Nuria J. Thusius, Teresa A. Rummans, Paul E. Croarkin, Victor M. Karpyak, Brian A. Lynch, Kathryn M. Schak

**Affiliations:** 1 Department of Psychiatry and Psychology, Mayo Clinic, 200 First St SW, Rochester, MN 55905 USA; 2 Division of Community Pediatric and Adolescent Medicine, Mayo Clinic, Rochester, MN USA; 3 Fountain Centers, Mayo Clinic Health System in Albert Lea, Albert Lea, MN USA

**Keywords:** Attachment, Child outcomes, Opioid use, Parent–child relationship, Substance use disorder

## Abstract

**Background:**

Between 2009 and 2014, nearly 3% of US children (age ≤ 17 years) lived in households with at least 1 parent with substance use disorder. The present systematic review aimed to evaluate effects of parental opioid use disorder on the parent–child relationship and child developmental and behavioral outcomes.

**Methods:**

Several databases were comprehensively searched for studies published from January 1980 through February 2018 that reviewed effects of parental opioid addiction on parent–child relationships and outcomes of children (age, 0–16 years).

**Results:**

Of 304 unique studies, 12 evaluated effects of parental opioid addiction on the parent–child relationship as the primary outcome and on children’s outcomes, including behaviors and development. Observation of mother–child interaction showed that mothers with opioid use disorders are more irritable, ambivalent, and disinterested while showing greater difficulty interpreting children’s cues compared with the control group. Children of parents with opioid use disorders showed greater disorganized attachment; they were less likely to seek contact and more avoidant than children in the control group. The children also had increased risk of emotional and behavioral issues, poor academic performance, and poor social skills. Younger children had increased risk of abuse or neglect, or both, that later in life may lead to such difficulties as unemployment, legal issues, and substance abuse.

**Conclusions:**

Current evidence shows association between parental opioid addiction and poorer mother–child attachment and suboptimal child developmental and behavioral outcomes. Further research and treatment targeting children and families with parental opioid use are needed to prevent difficulties later in life.

**Electronic supplementary material:**

The online version of this article (10.1186/s13034-019-0266-3) contains supplementary material, which is available to authorized users.

## Introduction

The National Survey on Drug Use and Health (NSDUH) reported that between 2009 and 2014, nearly 3% (2.1 million) of US children age 17 years and younger lived in households with at least 1 parent struggling with a substance use disorder [1]. Between 2009 and 2014, an annual average of 464,000 children younger than 2 years were cared for by at least 1 parent with illicit substance use. It is unclear how many of those parents were using prescribed or illicit opioids, or both [1]. However, the most recent NSDUH report estimated that 2 million persons in the United States abused prescription opioid pain medications and 591,000 were using heroin in 2015 [2].

The opioid epidemic is growing, and many users are women of childbearing age. A limited number of studies have examined pre- and perinatal influences of parental opioid use on childhood development and medical comorbidities [3–6]. Two studies reported that methadone use during pregnancy may affect children in motor development delays, vulnerability to life adversities for boys, and behavioral issues [3, 7]. However, Johnson and Rosen [3] described a group of mothers participating in a methadone treatment program whose children were overall relatively healthy (on the basis of physical and neurologic examinations and a battery of behavioral assessments). Regarding individual subgroups, sons of mothers in a methadone program had lower parent-directed interaction scores (i.e., predictors of developmental difficulties) than a comparison control group [3]. Even less is known about what happens to children exposed to caregivers with opioid use disorder during their developmental trajectory [8, 9].

In 2016, the US Department of Health and Human Services reported that foster care systems nationwide are having a marked increase in children requiring placement because of parental opioid abuse [10]. Families struggling with opioid addiction often have severe poverty, legal issues, and domestic violence, along with parental medical and psychiatric issues [11–13]. Children of parents with opioid use disorder may be at an increased risk for attention-deficit/hyperactivity disorder, learning difficulties, troubles at school, substance use disorders, and other mental health issues [14–16]. The 1998 Adverse Childhood Experience Study showed a correlation between the number of adverse childhood experiences (ACEs) and risk of substance abuse during adulthood [17]. Offspring of parents with opioid use disorder also have an increased incidence of childhood abuse and neglect and negative outcomes in adulthood [17].

The effects of parental opioid use on the parents’ children are still grossly unknown. In light of the opioid abuse epidemic, we believe it is relevant to review and critically evaluate the findings of studies that attempted to examine the effects of parental opioid use on attachment, parent–child relationship, and child developmental and behavioral outcomes. The primary aim of this systematic review was to summarize this research. The secondary aim was to identify knowledge gaps in the existing literature that could serve as a basis for future research. This body of research could then inform social policy changes.

## Methods

### Protocol and registration

This review was planned and conducted with the protocol published in the PROSPERO database, under No. CRD42016038470 and available at https://www.crd.york.ac.uk/PROSPERO [18]. The study closely followed guidelines of Preferred Reporting Items for Systematic Reviews and Meta-analyses (PRISMA) Moher et al. [19].

### Eligibility criteria

In accordance with Santos et al. [20] we used the acronym PICO—patient, intervention, comparison, and outcomes—to develop a study. We asked whether opioid use are a risk factor for poor parenting skills and low parental functioning that correlates with a poor parent–child relationship and difficult behaviors in children. We defined our “P” population as parents (1 parent or both) with opioid use disorder and their children. Most studies were observational (the “I”), and since a limited number of studies contained comparisons with control samples, we included studies that had no comparison group. The “O” was parent–child interactions, including attachment style, and the child’s developmental and behavioral outcomes, including mental health and school or work performance. Selection criteria included published articles written in English between January 1, 1980, and February 1, 2018. Our review contained cohort, case–control, cross-sectional, and ecological study designs.

**Fig. 1:**
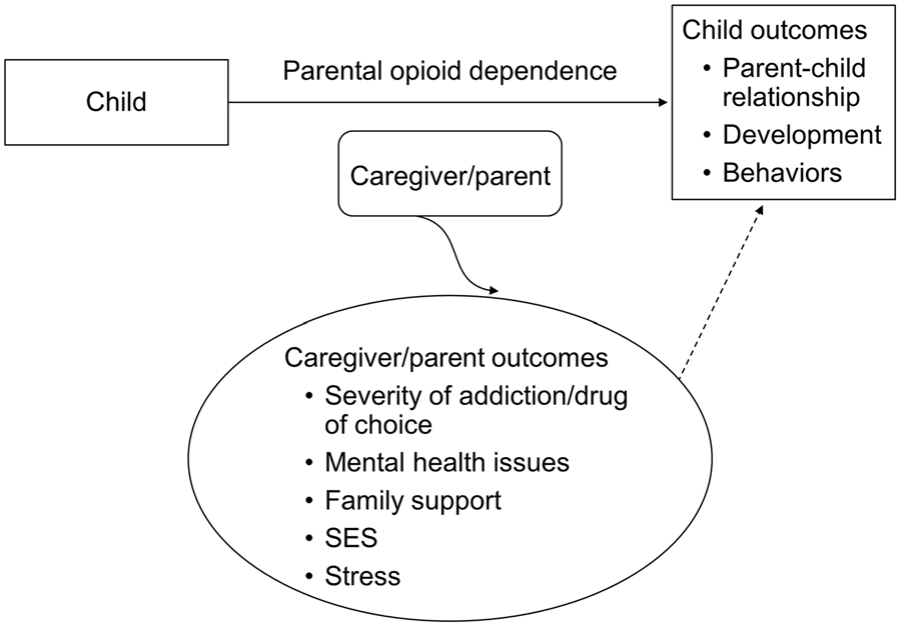
Analytic framework. Effects of parental opioid use on the parent–child relationship and the children’s developmental and behavioral outcomes. SES indicates socioeconomic status

Studies were excluded if participants were using a nonopioid substance, only alcohol, or polysubstances. Given that the recent opioid epidemic involves a new socioeconomic group (addiction to prescription medication), we included all types of opioids and did not limit studies to active abuse only (e.g., if parents were actively engaged in treatment program). We did not exclude studies that assessed effects of prenatal exposure as long as they also measured elements of parent–child interactions. We excluded studies that included only parental outcomes without comment on the children. We also excluded those that focused solely on prenatal effects of parental opioid use on children’s behavior. We primarily were interested in descriptions of outcomes for children ages 0 to 16 years that included neonates and infants. Figure 1 illustrates the analytic framework of this review.

### Search strategies and information sources

A comprehensive search of several databases was conducted and contained studies in the English language from the selected period. The databases included Ovid MEDLINE Epub Ahead of Print, Ovid Medline In-Process and Other Non-Indexed Citations, Ovid MEDLINE, Ovid EMBASE, Ovid PsycINFO, Ovid Cochrane Central Register of Controlled Trials, Ovid Cochrane Database of Systematic Reviews, and Scopus. An experienced librarian created and conducted the search strategy, with the principal investigator’s input. Keywords supplemented the controlled vocabulary in the search for the effects of parental opioid addiction on the parents’ children. Additional file 1: Appendix S1 shows the detailed strategy. The review protocol was registered in PROSPERO (No. CRD42018087539) on February 13, 2018. To ensure no studies were omitted, 2 authors (MR and JLVV) performed backward searches and reviewed the bibliographies of the included studies. The database search was inclusive to capture a wide range of studies, and it contained terms in 2 categories: parental opioid use (parent, mother, father, paternal, maternal, guardian, custodian AND opioids, heroin, methadone, morphine) and the parent–child relationship (e.g., parenting, observational methods, parent–child interaction, mother–child relations, parenting style observation methods, attachment, bonding).

### Study selection and data extraction process

The selected studies that used above-mentioned search criteria were entered into the EndNote ×6 (Thomson Reuters), and duplicates were eliminated. Articles were excluded if their abstracts were not available or they were not in the English language. Two authors (MR and JLVV) reviewed titles and abstracts for study inclusion. If these 2 reviewers disagreed on study inclusion, then a third author (KMS) made the final decision. If it was unclear which substance was used by the parents, the full-text study was examined for eligibility. Two authors (MR and JLVV) reviewed the full text of manuscripts identified through title and abstract screening, and any that met exclusion criteria were eliminated.

We collected the following information on each study: year, site, sample size, period, description of person exposed to opioids and addiction history, presence or absence of control group, age of child at outcome, presence or absence of follow-up information for children, statistical analyses, study focus, types of assessment methods used for children and parents, and main findings in regard to parent–child interactions and child behaviors and development.

### Quality evaluation of the studies

Given the heterogeneity of the study data, the summary of results was quantitative and descriptive and had no meta-analysis. With the small number of included studies and the descriptive nature of this review, we were unable to perform meta-analysis or any additional test such as sensitivity analysis, selective reporting, or publication bias.

## Results

**Fig. 2:**
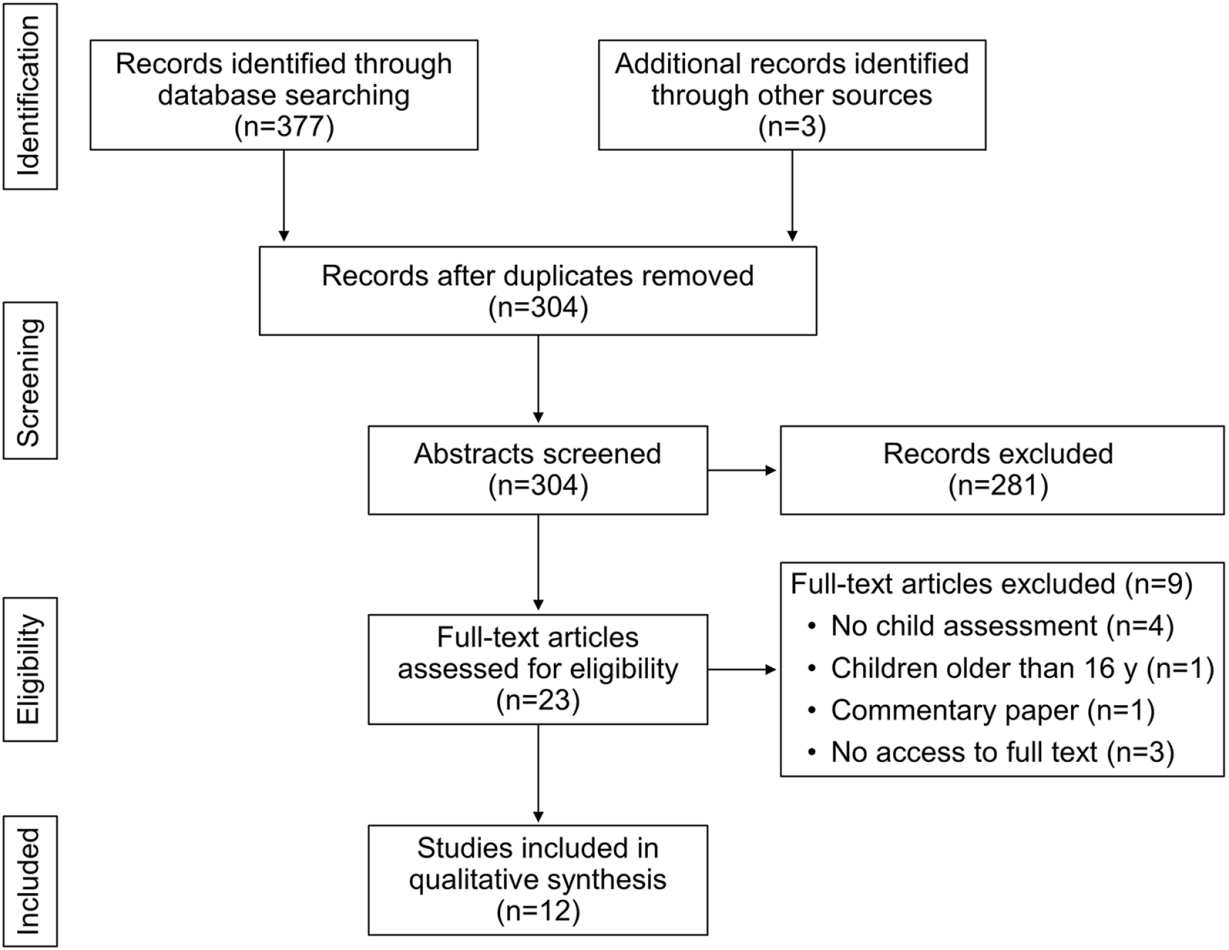
Summary of included and excluded studies. Summary based on adapted Preferred Reporting Items for Systematic Reviews and Meta-analysis (PRISMA) 2009 flow diagram

Figure 2 [21] outlines the article selection process. The initial search identified 380 studies, with 377 from the search of selected databases and 3 added from the reference lists of the selected articles. After removal of duplicates, 304 potentially qualifying abstracts were reviewed; 281 were excluded because they did not meet inclusion criteria. Reasons for exclusion were primarily based on opioid abuse as a part of polysubstance use without clear differentiation, lack of focus on parent–child relationship, and focus only on children or only on parents. In total, 23 articles were selected to further examine in full text for eligibility. Of those, 9 studies were removed (4 did not include information on child assessment, 1 included only children older than 16 years, 1 was a commentary, and 3 had no access to full text). This latest exclusion left 12 studies that met the full range of inclusion and exclusion criteria.

### Study characteristics

Of 12 studies that involved mothers with opioid use disorder (e.g., heroin, prescription opioids) and who were not using any other drugs [22–33] (Table 1), 9 evaluated mothers participating in methadone maintenance programs [22–28, 30–33], 2 had mothers from methadone and buprenorphine clinics [31, 32], and 1 had mothers receiving buprenorphine replacement therapy [29]. All but 1 study included mothers only [22–27, 29–33]. The study by Skinner et al. [28] included mothers and fathers as primary caregivers. Children’s ages ranged from 0 to 16 years, and 75% of the studies (n = 8) focused on infancy and toddlerhood years [23–25, 27, 29, 31–33]. Only 1 study was longitudinal, and it monitored families over 14 years [28]. A study by Borelli et al. [30] was cross-sectional and part of larger randomized clinical trial.

**Table 1:** Summary of studies that evaluated parents with opioid use disorder and no other drug use

Study/type	Sample size	Comparison group	Exposure by whom	Outcome at age, mother’s or child’s	Study focus	Findings
[22] /comparative–descriptive study	15 mothers receiving MM and their 15 preschool children	15 NDA mothers and their 15 preschool children	Mothers	Screening session; sessions 1 and 2 at 2-weeks intervals. Children’s mean age for MM group, 4.3 years; control mean age, 3.8 years	Drug Abuse Treatment Referral System; California Psychological Inventory; Parental Attitudes Questionnaire; Minnesota Child Development Inventory; Stanford-Binet Intelligence Scale; Motor Scale of the McCarthy Scales of Children’s Abilities; videotaped mother–child interaction during 2 play sessions using the Interactional Coding System	MM mothers struggled with their parenting behaviors in comparison to controls. No differences were noted in parenting attitudes. Children of MM mothers had lower measures of intelligence than controls
[23] /comparative–descriptive study	17 methadone-exposed infants	23 same-age comparison infants	Mothers	4 months	Communicative functioning in dyadic interaction, motor functioning in infants	Mothers with poor interactive skills were likely to have poor maternal resources, and their infants were at increased risk for low communicative functioning and problematic motor functioning. Methadone use was 1 of the risk factors affecting interaction
[24] /comparative–descriptive study	30 methadone-exposed toddlers	44 comparison toddlers	Mothers	24 months	Mother was instructed to teach the child how to use a toy (focused attention)	No difference in focused attention based only on prenatal exposure
[25] /comparative–descriptive study	35 methadone-exposed infants	46 comparison infants	Mothers	12 months	Separation–reunion procedure, mothers’ perceptions during pregnancy of how bothersome their children will be	Less contact-maintaining behavior than controls but similar proximity-seeking behaviors between the two groups. High perceptions of bothersomeness were related to higher scores on contact-maintaining behavior and lower scores on avoidant behavior
[26] /comparative–descriptive study	69 mothers with opioid use disorder participating in methadone treatment with children < 16 years	51 SES-matched comparison mothers	Mothers	Baseline assessment children’s mean (SD) age for opiate group, 10.0 (3.9) years; control group, 9.6 (4.6) years	BASC; Parent–Child Relationship Inventory	SES and how mothers perceive their children’s difficult behaviors have a mediating role in parenting strategies for mothers with opioid use disorder. Authors concluded that the level of parental involvement can be directly linked to addiction issues
[27] /comparative–descriptive study	30 toddlers exposed in utero to methadone	42 comparison toddlers	Mothers	12 and 24 months	Infant attachment behaviors at 12 months, mother and child communication at 24 months, mothers’ perceptions during pregnancy of their children’s bothersomeness	No direct correlation between use of methadone and quality of communication between mother and child, correlation was moderated by mother’s idea of infant’s bothersomeness and mediated by infant’s avoidant attachment style
[28] /longitudinal study	144 parents from methadone clinic and 177 children	No comparison group	Mothers and fathers	Children between the ages of 3 and 15 years at baseline and then 6, 12, and 24 months following the intervention. Follow-up interview 14 years later	ACEs; parent recovery, parent–child bonding, and family management were measured on basis of child report scales	A high likelihood of ACEs and only 3% of children had no other risks than having a parent with opioid use disorder. Of the young adults, 24% met all criteria for functional resilience. The earlier there were behavioral problems, the more significantly reduced were the chances of functional resilience
[29] /comparative–descriptive study	15 opioid-exposed participants in buprenorphine replacement therapy program	15 maternal depression infant dyads and 57 unexposed mother–infant dyads	Mothers	Second half of first year	Bayley MDI; Emotion Availability Scales, 3rd edition	Opioid-exposed infants had lowest Bayley-II MDI scores and scored lowest in infant involvement. Opioid-abusing mothers had lowest scores in maternal sensitivity, structuring, and nonintrusiveness. There were more separations from mothers with opioid use disorders in end of first year for their infants and higher risk of physical abuse
[30] /cross-sectional study, part of a larger RCT	99 mothers receiving MM and their children	None	Mothers	Baseline assessment for RCT (children age, 4–16 years)	Parent and child versions of Parental Acceptance-Rejection Questionnaire; BASC; Beck Depression Inventory for maternal depression	Mothers noted themselves to be less aggressive than their own children’s view of them. Children tended to report both internalizing and externalizing behaviors; their mothers reported only children’s externalizing problems
[31] /comparative–descriptive study	33 heroin-using mothers in opioid maintenance treatment (methadone or buprenorphine) and their toddlers	36 mothers and toddlers in comparison group	Mothers	Last trimester of pregnancy. After delivery, assessment every third month until 12 months. Follow-up at toddler age 2.5 years	Parenting stress index, long version; Hopkins Symptom Checklist-25; videotaped semi-structured play that followed NICHD Study of Early Child Care guidelines; child behavior checklist; Pediatric Quality of Life Inventory (PedsQL; Mapi Research Trust) 4.0	Toddlers in group with opioid use disorders had significantly more perceived problems. After control for factors that could be responsible for developmental outcomes, opioid exposure was not independently responsible for parent–child interaction issues
[32] /comparative–descriptive study	33 heroin-using mothers in opioid maintenance treatment program (methadone or buprenorphine) and their toddlers	36 mothers and toddlers comparison group	Mothers	6 months	Videotaped semistructured play that followed NICHD Study of Early Child Care guidelines	Maternal style that consists of 6 maternal behaviors during free-play was significantly responsible for “dyadic mutuality.” Other factors such as maternal drug use before opioid treatment program, maternal depression, level of parenting stress, and developmental issues of infants significantly affected dyadic mutuality
[33] /comparative–descriptive study	12 opiate-exposed mother-infant dyads from methadone treatment program	33 comparison infants < 1 month old	Mothers	One-time 30-min recording during feeding at age 0–1 month	Nursing Child Assessment Satellite-Training Scale scores	Opiate-exposed dyads scored significantly lower in infant subscales of clarity of cues, responsiveness to caregiver, and total score than comparison sample. Parents showed lower sensitivity to infant cues and their contingency score was also significantly lower

*ACE* adverse childhood experience, *BASC* Behavioral Assessment System for Children, *MDI* Mental Development Index, *MM* methadone maintenance, *NDA* non-drug-addicted, *NICHD* National Institute of Child Health and Human Development, *RCT* randomized clinical trial, *SES* socioeconomic status

### Effects of parental opioid use on attachment

Of note, only 2 studies by Goodman et al. in 1999 [25] and in 2005 [27] directly addressed attachment style in the children of parents with opioid use disorder who were receiving methadone treatment. Both studies assessed the infants’ attachment at 12 months through the Strange Situation paradigm [34], and Goodman et al. [27] assessed mother–child communication at 24 months (videotaped for 40 min) [35]. Goodman et al. [25] found that children born to mothers with opioid use disorder through the Strange Situation paradigm showed increased levels of disorganized attachment at 12 months. They were less likely to seek contact and were more avoidant than the control group.

The study assessed other uses external from methadone use variables for mediation between exposure to methadone and attachment style. The main variables were annual per capita income for mother’s household, anticipated difficulties of infant’s behaviors perceived by the mother during pregnancy (authors labeled it as *bothersomness*), anticipated difficulties in the infant’s behaviors perceived by the mother during pregnancy in comparison to regular infant, and parity (number of children to whom the mother gave birth). The variables did not show mediating relations between opioid use and indexes of attachment measures. The authors speculated that perhaps neurologic pathways that were not addressed in their study have a mediating relationship between methadone exposure and insecure attachment style.

Goodman et al. [27] did a another study where they examined relation between exposure to methadone and mother–child relationship measured on the basis of the separation–reunion situation at 12 months and 8 scripted and videotaped situations at 24 months. The study authors hypothesized that the mothers’ and infants’ affect organization and their communication style may affect how methadone exposure influences a mother’s communication style. Anticipated difficulties of an infant’s behaviors perceived by the mother during pregnancy turned out to have a moderating effect on methadone exposure on the quality of communication between the mother and her baby. This may have potential implications for treatment programs because it would suggest the importance for mothers and their infants to receive part of the treatment together to help with their relationship early on.

### Effects of parental opioid use on parenting style and parent–child relationship

Seven observational studies directly looked at parent–child relationship and provided information on parenting style and child responsiveness [22–24, 27, 31–33]. Four studies used only assessment scales that allowed for indirect assessment of parent–child relationship [26, 28–30].

Bauman and Dougherty [22] compared 15 mothers of preschool children receiving methadone maintenance (MM) to the same number of non-drug-addicted mothers and their children. The investigators found no difference between groups in parenting attitudes. However, the mothers with opioid use disorder were markedly less adaptive in their parenting behaviors. MM mothers were noted to be more critical and more negative, and at times even threatening, toward their children. A longitudinal study by Jeremy and Bernstein [23] rated maternal communicative functioning and quality of interactions between mothers and their 4-month-old infants. Participant dyads that struggled with their relationship were more at risk for presenting with problematic behaviors. The authors concluded that methadone use was an independent risk factor, acting along with the parent–child relationship quality.

Maternal psychological well-being also has an important impact on parenting ability. Sarfi et al. [32] studied videotaped interactions between mothers in opioid maintenance treatment and their 6-month-old infants. The videotapes showed that the quality of the mother-infant relationship was strongly associated with maternal depression, parenting stress, and their infants’ level of development; yet, opioid use was not an independent risk factor for difficult parent–child relationships. In contrast, Suchman and Luthar [26] found a direct link between maternal opioid addiction and lack of parental involvement. They noted ambivalence and frequent lack of involvement and initiative among mothers in the context of raising their children. This finding was confirmed by Salo et al. [29], who identified that opioid-abusing mothers struggled with maternal sensitivity, structuring, and nonintrusiveness. The authors also described a well-known phenomenon of parents’ desire to “farm out” their children to friends and relatives, and they noted a higher number of separations between mothers with opioid use disorder and infants during the first year of life.

In an observational study by Maguire et al. [33], dyads were recorded for 30 min during the feeding of infants 0 to 1 month of age while in the hospital. Compared with control infants, the infants of mothers with opioid use disorder struggled with clarity of cues and responsiveness to a caregiver and received lower total scores on the Nursing Child Assessment Satellite-Training Scale [36]. In addition, mothers with opioid use disorder showed less sensitivity to infants’ cues.

### Children’s developmental and behavioral outcomes

Eight sets of study authors strived to address whether opioid use was a single independent risk factor for a child’s difficulties or whether an association existed between parental drug use and outcomes such as low socioeconomic status (SES); poverty; history of abuse or neglect, or both; parental unemployment; and poor nutrition [23–27, 31–33]. One study found SES and maternal perception, irrespective of opioid use [26], as factors that mediated parenting strategies. Ironically, 2 other maternal perception studies found that when a mother perceived her infant as more burdensome, the parent–child relationship had fewer struggles and the child presented with less difficult behaviors [25, 27]. Study authors interpreted that if mothers were more aware of their negative feelings, they were less likely to act negatively toward their children, which aided in attachment security. Of note, only 1 study included fathers, and this lack of inclusion of fathers appears to be a limitation of existing research [28]. Only 2 studies found methadone use to be only one of the risk factors for child behavioral issues and attachment difficulties, independent of the parent–child relationship quality [23, 24]. Additionally, 1 study that examined functional resilience found that 25% of children exposed to parental drug use did not seem affected by their own legal issues and educational difficulties or substance use [28].

In the study of preschool children of mothers receiving MM [22], children were noted to be more hyperactive and disruptive than control students. They also had lower scores of intelligence and socially adaptive behaviors and tended to be more developmentally behind than control children. Another study [23] of 4-month-old infants showed that infants of mothers with lower communicative skills had worse motor coordination and greater motor tension. Schneider and Hans [24] investigated the impact of prenatal opioid use on attention in 2-year-old toddlers and found no difference in focused attention between the children of mothers with opioid use disorder and the control children.

Another study, by Borelli et al. [30] investigated older children (age 4–16 years) and found that mothers reported a marked number of both internalizing and externalizing behaviors in the children.

Skinner et al. [28] took a different approach than the prior investigators discussed herein. They researched factors contributing to resilience in the children of parents with opioid use disorder. They evaluated children and their families while parents participated in an MM program and then invited participants for a follow-up interview 14 years later. On follow-up, 70% of the young adults reported having at least 2 additional ACEs, and 20% had at least 4 ACEs. Only 24% of interviewees met criteria for functional resilience, which authors defined as working or going to school, no substance use, and no legal history in the past 5 years.

We have included a broad age range of children in our literature search because we believed that opioid addiction would affect parenting behaviors and child outcomes at different ages depending on the child’s age during which the caregiver struggled with opioid addiction issues. Unfortunately, because of the limited number of studies that our search was able to retrieve, we were unable to answer this question.

## Discussion

To our knowledge, this is the first attempt to systematically summarize data on how parental opioid use affects the parent–child relationship and children’s developmental and behavioral outcomes. Despite the growing US opioid epidemic, a limited number of studies have been conducted on this major public health crisis. While negative prenatal effects of maternal opioid use are well understood, the long-term effects of parental opioid use and the dependence on the parent–child relationship and child development continue to be unknown.

We can examine the findings from three different points of view: parents (mothers) alone, children alone, and a parent–child dyad. Direct observation of mother–child interaction studies have found that mothers with opioid use disorder, in comparison to controls, are more irritable, ambivalent, and disinterested while exhibiting greater difficulty in interpreting their children’s cues. They also are less adaptive when it comes to parenting behaviors. For children age 3 to 4 years and older, mothers perceive their children as having many externalizing issues and fail to notice the internalizing ones. Consensus is lacking when it comes to mediating factors. Some studies suggest that methadone use serves as an independent risk factor; others list maternal psychological well-being, SES, and maternal attention as significantly and irrespectively impacting parenting ability.

Children of parents with opioid use disorders have more mental health issues than control children. However, although a number of studies list parental methadone use as an independent risk factor, others discuss as mediating factors the characteristics of low SES; history of abuse or neglect, or both; parental unemployment; and poor nutrition. For these reasons, upcoming research necessitates further clarification.

In terms of the parent–child relationship, children of parents with opioid use disorders show greater disorganized attachment, they are less likely to seek contact, and they are more avoidant than the control children. Although this finding is based on a limited number of studies, it is worrisome and should be investigated further because of its importance for policy making and programming.

### Gaps in the literature and limitations

An important strength of this review is all the studies having a control group or addressing confounding factors in their analyses. Numerous literature gaps exist in how parental opioid use effects the parent–child relationship and children’s development. First, few studies have examined this topic especially in the current environment of increased opioid use. Second, most of the studies had a small number of participants. Third, the history of parental drug use was poorly recorded and often unclear, making it difficult to assess correlations among types of opioids used, dose, and length of use and the effects on the parent–child relationship and child behaviors. Fourth, although opioid consumption is now an epidemic across all SES groups, most studies recruited from low SES and excluded the impact of opioid use in groups with higher SES. Fifth, there is a paucity of controlled longitudinal studies that could better discern causality. Sixth, most studies had participants recruited from methadone or suboxone maintenance programs, or both (i.e., active receipt of opioid addiction treatment). For ethical and legal reasons, it would be difficult to include parents addicted to heroin or other illicit substances; yet those families may be most in need of interventions. Future studies should include those populations through recruitment from emergency departments or treatment programs that particularly target heroin users. Future research also should evaluate the effect of parental opioid addition on nonaddicted members of the family. Additionally, more knowledge is needed about the roles of fathers or other extended family members in influencing the association of maternal opioid addiction and childhood developmental and behavioral outcomes.

Many efforts are under way to address the opioid crisis; however, they will be effective only if the medical community provides treatment that targets the family systems and units involved. Unfortunately, simply reducing the number of available drugs is not enough. Children of parents with opioid use disorders are a population at risk and need special care. The medical community needs policies in place that support efforts to prevent opioid addiction at a young age for children who were exposed to it through their parents.

## Conclusions

Our review of the existing literature indicates that parental opioid dependence affects the parent–child relationship, child development, and child behaviors. Although many ethical and legal factors need to be considered when studying such a vulnerable population, prospective cohort studies that allow further analysis of associations between parental opioid use and effects on the parent–child relationship are feasible and needed. Given the current US opioid epidemic, the need is clear for additional research that targets children of parents with opioid use disorders and may support child and family interventions.

## Additional file

**Additional file 1.** Appendix S1.

## Abbreviations

ACE: adverse childhood experience
MM: methadone maintenance
NSDUH: National Survey on Drug Use and Health
PICO: patient, intervention, comparison, and outcomes
PRISMA: Preferred Reporting Items for Systematic Reviews and Meta-analyses
SES: socioeconomic status
