# Supplementary material for: The effects of parental opioid use on the parent–child relationship and children’s developmental and behavioral outcomes: a systematic review of published reports
Source: Child Adolesc Psychiatry Ment Health. 2019 Jan 12;13:5. doi: 10.1186/s13034-019-0266-3 (PMC6330457; doi:10.1186/s13034-019-0266-3)
Supplement: Supplementary file 1 — Additional file 1. Appendix S1. [file 13034_2019_266_MOESM1_ESM.doc]

Appendix S1

Ovid

Database(s): Embase 1988 to 2018 Week 05, PsycINFO 1806 to January Week 4 2018, EBM Reviews - Cochrane Central Register of Controlled Trials December 2017, EBM Reviews - Cochrane Database of Systematic Reviews 2005 to January 31, 2018, Ovid MEDLINE(R) Epub Ahead of Print, In-Process & Other Non-Indexed Citations, Ovid MEDLINE(R) Daily and Ovid MEDLINE(R) 1946 to Present
Search Strategy:

| **#** | **Searches** | **Results** |
| --- | --- | --- |
| 1 | exp Opioid-Related Disorders/ | 37484 |
| 2 | ((acetorphine or acetylcodeine or acetylmethadol or Alfentanil or Alphaprodine or anileridine or apadoline or azidomorphine or benzhydrocodone or bezitramide or bremazocine or "Brompton mixture" or Buprenorphine or Butorphanol or ciramadol or cocodamol or Codeine or codydramol or conorfone or cyclazocine or Dextromoramide or Dextropropoxyphene or dextrorphan or dezocine or diamorphine or diconal or dihydrocodeine or dihydroetorphine or Dihydromorphine or dimethylthiambutene or Diphenoxylate or dipipanone or enadoline or eptazocine or ethylketazocine or Ethylketocyclazocine or Ethylmorphine or etonitazene or Etorphine or etoxeridine or faxeladol or Fentanyl or furethidine or gelonida or Heroin or Hydrocodone or isalmadol or isomethadone or ketazocine or ketobemidone or ketogan or kyotorphin or lefetamine or levacetylmethadol or levomethadone or Levorphanol or Meperidine or Meptazinol or metazocine or Methadone or "Methadyl Acetate" or methylsamidorphan or Morphine or "morphinomimetic agent*" or "morphinomimetic drug*" or morphinone or Nalbuphine or narcotic* or nicocodine or nicomorphine or noracymethadol or norbuprenorphine or nordextropropoxyphene or normorphine or norpethidine or norpropoxyphene or "o nortramadol" or oliceridine or opiate or Opiate* or opioid* or Opium or oripavine or Oxycodone or Oxymorphone or pentamorphone or Pentazocine or pethidine or phenadoxone or phenaridine or Phenazocine or phencyclidine or Phenoperidine or picenadol or piminodine or Pirinitramide or piritramide or profadol or Promedol or propiram or sameridine or samidorphan or semorphone or Sufentanil or tapentadol or thebaine or tifluadom or Tilidine or tonazocine or Tramadol or trimeperidine) adj3 (abuse or abuse* or abusing or addict* or addiction* or dependenc* or disorder* or habit* or misusage or misuse or misusing or toxicomania* or toxicomanie)).ti,ab,hw,kw. | 77233 |
| 3 | 1 or 2 | 77233 |
| 4 | exp Parents/ | 377289 |
| 5 | (father or fathers or maternal or mother or mothers or parent or parental or parents or paternal or pregnan* or stepparent or stepparents or "Surrogate Mother*").ti,ab,hw,kw. | 2972742 |
| 6 | 4 or 5 | 2972899 |
| 7 | exp "Child of Impaired Parents"/ | 5046 |
| 8 | (newborn* or neonat* or infant* or toddler* or child* or adolescent* or paediatric* or pediatric* or girl or girls or boy or boys or teen or teens or teenager* or preschooler* or "pre-schooler*" or preteen or preteens or "pre-teen" or "pre-teens" or youth or youths or offspring).ti,ab,hw,kw. | 8184751 |
| 9 | 7 or 8 | 8184751 |
| 10 | 3 and 6 and 9 | 3364 |
| 11 | limit 10 to english language [Limit not valid in CDSR; records were retained] | 3053 |
| 12 | limit 11 to yr="1980 -Current" | 2810 |
| 13 | (exp animals/ or exp nonhuman/) not exp humans/ | 8973071 |
| 14 | ((alpaca or alpacas or amphibian or amphibians or animal or animals or antelope or armadillo or armadillos or avian or baboon or baboons or beagle or beagles or bee or bees or bird or birds or bison or bovine or buffalo or buffaloes or buffalos or "c elegans" or "Caenorhabditis elegans" or camel or camels or canine or canines or carp or cats or cattle or chick or chicken or chickens or chicks or chimp or chimpanze or chimpanzees or chimps or cow or cows or "D melanogaster" or "dairy calf" or "dairy calves" or deer or dog or dogs or donkey or donkeys or drosophila or "Drosophila melanogaster" or duck or duckling or ducklings or ducks or equid or equids or equine or equines or feline or felines or ferret or ferrets or finch or finches or fish or flatworm or flatworms or fox or foxes or frog or frogs or "fruit flies" or "fruit fly" or "G mellonella" or "Galleria mellonella" or geese or gerbil or gerbils or goat or goats or goose or gorilla or gorillas or hamster or hamsters or hare or hares or heifer or heifers or horse or horses or insect or insects or jellyfish or kangaroo or kangaroos or kitten or kittens or lagomorph or lagomorphs or lamb or lambs or llama or llamas or macaque or macaques or macaw or macaws or marmoset or marmosets or mice or minipig or minipigs or mink or minks or monkey or monkeys or mouse or mule or mules or nematode or nematodes or octopus or octopuses or orangutan or "orang-utan" or orangutans or "orang-utans" or oxen or parrot or parrots or pig or pigeon or pigeons or piglet or piglets or pigs or porcine or primate or primates or quail or rabbit or rabbits or rat or rats or reptile or reptiles or rodent or rodents or ruminant or ruminants or salmon or sheep or shrimp or slug or slugs or swine or tamarin or tamarins or toad or toads or trout or urchin or urchins or vole or voles or waxworm or waxworms or worm or worms or xenopus or "zebra fish" or zebrafish) not (human or humans or patient or patients)).ti,ab,hw,kw. | 8055717 |
| 15 | 12 not (13 or 14) | 2628 |
| 16 | exp Parent-Child Relations/ | 179574 |
| 17 | ((father or fathers or maternal or mother or mothers or parent or parental or parents or paternal or pregnan* or stepparent or stepparents or "Surrogate Mother*") adj5 (newborn* or neonat* or infant* or toddler* or child* or adolescent* or paediatric* or pediatric* or girl or girls or boy or boys or teen or teens or teenager* or preschooler* or "pre-schooler*" or preteen or preteens or "pre-teen" or "pre-teens" or youth or youths or offspring) adj5 (attach* or attitud* or bond* or correlation* or influenc* or interaction* or relation* or "spatial pattern*")).ti,ab,hw,kw. | 212378 |
| 18 | 16 or 17 | 231880 |
| 19 | 15 and 18 | 377 |
| 20 | remove duplicates from 19 | 276 |

Scopus

1 TITLE-ABS-KEY((acetorphine or acetylcodeine or acetylmethadol or Alfentanil or Alphaprodine or anileridine or apadoline or azidomorphine or benzhydrocodone or bezitramide or bremazocine or "Brompton mixture" or Buprenorphine or Butorphanol or ciramadol or cocodamol or Codeine or codydramol or conorfone or cyclazocine or Dextromoramide or Dextropropoxyphene or dextrorphan or dezocine or diamorphine or diconal or dihydrocodeine or dihydroetorphine or Dihydromorphine or dimethylthiambutene or Diphenoxylate or dipipanone or enadoline or eptazocine or ethylketazocine or Ethylketocyclazocine or Ethylmorphine or etonitazene or Etorphine or etoxeridine or faxeladol or Fentanyl or furethidine or gelonida or Heroin or Hydrocodone or isalmadol or isomethadone or ketazocine or ketobemidone or ketogan or kyotorphin or lefetamine or levacetylmethadol or levomethadone or Levorphanol or Meperidine or Meptazinol or metazocine or Methadone or "Methadyl Acetate" or methylsamidorphan or Morphine or "morphinomimetic agent*" or "morphinomimetic drug*" or morphinone or Nalbuphine or narcotic* or nicocodine or nicomorphine or noracymethadol or norbuprenorphine or nordextropropoxyphene or normorphine or norpethidine or norpropoxyphene or "o nortramadol" or oliceridine or opiate or Opiate* or opioid* or Opium or oripavine or Oxycodone or Oxymorphone or pentamorphone or Pentazocine or pethidine or phenadoxone or phenaridine or Phenazocine or phencyclidine or Phenoperidine or picenadol or piminodine or Pirinitramide or piritramide or profadol or Promedol or propiram or sameridine or samidorphan or semorphone or Sufentanil or tapentadol or thebaine or tifluadom or Tilidine or tonazocine or Tramadol or trimeperidine) W/3 (abuse or abuse* or abusing or addict* or addiction* or dependenc* or disorder* or habit* or misusage or misuse or misusing or toxicomania* or toxicomanie))

2 TITLE-ABS-KEY ( ( father OR fathers OR maternal OR mother OR mothers OR parent OR parental OR parents OR paternal OR pregnan* OR stepparent OR stepparents OR "Surrogate Mother*" ) W/5 ( newborn* OR neonat* OR infant* OR toddler* OR child* OR adolescent* OR paediatric* OR pediatric* OR girl OR girls OR boy OR boys OR teen OR teens OR teenager* OR preschooler* OR "pre-schooler*" OR preteen OR preteens OR "pre-teen" OR "pre-teens" OR youth OR youths OR offspring ) W/5 ( attach* OR attitud* OR bond* OR correlation* OR influenc* OR interaction* OR relation* OR "spatial pattern*" ) )

3 PUBYEAR AFT 1979 AND LANGUAGE(english)

4 1 and 2 and 3

5 TITLE-ABS-KEY((alpaca OR alpacas OR amphibian OR amphibians OR animal OR animals OR antelope OR armadillo OR armadillos OR avian OR baboon OR baboons OR beagle OR beagles OR bee OR bees OR bird OR birds OR bison OR bovine OR buffalo OR buffaloes OR buffalos OR "c elegans" OR "Caenorhabditis elegans" OR camel OR camels OR canine OR canines OR carp OR cats OR cattle OR chick OR chicken OR chickens OR chicks OR chimp OR chimpanze OR chimpanzees OR chimps OR cow OR cows OR "D melanogaster" OR "dairy calf" OR "dairy calves" OR deer OR dog OR dogs OR donkey OR donkeys OR drosophila OR "Drosophila melanogaster" OR duck OR duckling OR ducklings OR ducks OR equid OR equids OR equine OR equines OR feline OR felines OR ferret OR ferrets OR finch OR finches OR fish OR flatworm OR flatworms OR fox OR foxes OR frog OR frogs OR "fruit flies" OR "fruit fly" OR "G mellonella" OR "Galleria mellonella" OR geese OR gerbil OR gerbils OR goat OR goats OR goose OR gorilla OR gorillas OR hamster OR hamsters OR hare OR hares OR heifer OR heifers OR horse OR horses OR insect OR insects OR jellyfish OR kangaroo OR kangaroos OR kitten OR kittens OR lagomorph OR lagomorphs OR lamb OR lambs OR llama OR llamas OR macaque OR macaques OR macaw OR macaws OR marmoset OR marmosets OR mice OR minipig OR minipigs OR mink OR minks OR monkey OR monkeys OR mouse OR mule OR mules OR nematode OR nematodes OR octopus OR octopuses OR orangutan OR "orang-utan" OR orangutans OR "orang-utans" OR oxen OR parrot OR parrots OR pig OR pigeon OR pigeons OR piglet OR piglets OR pigs OR porcine OR primate OR primates OR quail OR rabbit OR rabbits OR rat OR rats OR reptile OR reptiles OR rodent OR rodents OR ruminant OR ruminants OR salmon OR sheep OR shrimp OR slug OR slugs OR swine OR tamarin OR tamarins OR toad OR toads OR trout OR urchin OR urchins OR vole OR voles OR waxworm OR waxworms OR worm OR worms OR xenopus OR "zebra fish" OR zebrafish) AND NOT (human OR humans or patient or patients))

6 4 and not 5

7 PMID(0*) OR PMID(1*) OR PMID(2*) OR PMID(3*) OR PMID(4*) OR PMID(5*) OR PMID(6*) OR PMID(7*) OR PMID(8*) OR PMID(9*)

8 6 and not 7
